# Epigenetic changes in blood leukocytes following an omega-3 fatty acid supplementation

**DOI:** 10.1186/s13148-017-0345-3

**Published:** 2017-04-26

**Authors:** Bénédicte L. Tremblay, Frédéric Guénard, Iwona Rudkowska, Simone Lemieux, Patrick Couture, Marie-Claude Vohl

**Affiliations:** 1 Institute of Nutrition and Functional Foods (INAF), Laval University, 2440 Hochelaga Blvd, Quebec, QC G1V 0A6 Canada; 2 CHU de Québec Research Center – Endocrinology and Nephrology, 2705 Laurier Blvd, Quebec, QC G1V 4G2 Canada

**Keywords:** DNA methylation, Omega-3 fatty acids, Microarray, Metabolic pathways, Blood leukocytes

## Abstract

**Background:**

Omega-3 polyunsaturated fatty acids (n-3 FAs) have several beneficial effects on cardiovascular (CV) disease risk factors. These effects on CV risk profile may be mediated by several factors, including epigenetic modifications. Our objective is to investigate, using genome-wide DNA methylation analyses, methylation changes following an n-3 FA supplementation in overweight and obese subjects and to identify specific biological pathways potentially altered by the supplementation.

**Results:**

Blood leukocytes genome-wide DNA methylation profiles of 36 overweight and obese subjects before and after a 6-week supplementation with 3 g of n-3 FAs were compared using GenomeStudio software. After supplementation, 308 CpG sites, assigned to 231 genes, were differentially methylated (FDR-corrected Diffscore ≥│13│~ *P* ≤ 0.05). Using Ingenuity Pathway Analysis system, a total of 55 pathways were significantly overrepresented following supplementation. Among these pathways, 16 were related to inflammatory and immune response, lipid metabolism, type 2 diabetes, and cardiovascular signaling. Changes in methylation levels of CpG sites within *AKT3*, *ATF1*, *HDAC4*, and *IGFBP5* were correlated with changes in plasma triglyceride and glucose levels as well as with changes in the ratio of total cholesterol/HDL-cholesterol following the supplementation.

**Conclusions:**

These data provide key differences in blood leukocytes DNA methylation profiles of subjects following an n-3 FA supplementation, which brings new, potential insights on metabolic pathways underlying the effects of n-3 FAs on CV health.

**Electronic supplementary material:**

The online version of this article (doi:10.1186/s13148-017-0345-3) contains supplementary material, which is available to authorized users.

## Background

Fish-oil-derived long-chain omega-3 fatty acids (n-3 FAs), including eicosapentaenoic acid (EPA, 20:5 n-3) and docosahexaenoic acid (DHA, 22:6 n-3) have several benefits on cardiovascular (CV) health. They exert hypotriglyceridemic [1, 2], anti-inflammatory [3–5], anti-arrhythmic [6, 7], and anti-thrombotic effects [8, 9]. Many factors, including genetic and epigenetic factors, may contribute to the observed effects of n-3 FAs on the CV risk profile. Indeed, emerging evidence suggests that n-3 FAs might influence global DNA methylation patterns due to their role in one-carbon metabolism [10]. A study in rats fed on a vitamin B_12_-deficient diet demonstrated that DHA modify DNA methylation, indicating that it plays a role in one-carbon metabolism [11]. DNA methylation is the best-characterized epigenetic factor and consists of the methylation of cytosine residues, mainly at cytosine-phosphate-guanine (CpG) dinucleotides [12]. The modification of DNA methylation by the environment may influence the regulation of CV risk factors, such as hypertension [13], atherosclerosis [14, 15], and inflammation [16]. Moreover, the methylation of repetitive sequences in blood has also been associated with CV diseases in epidemiological studies [17, 18].

Only a few studies have dealt with the impact of n-3 FAs on DNA methylation in human subjects. These studies were conducted in various populations including children and adolescents [19, 20], adults with renal impairment [21], women under caloric restriction [22], and Yup’ik Alaska Native individuals [23]. Moreover, a recent study demonstrated that a DHA supplementation during pregnancy was associated with changes in global methylation levels of inflammatory mediated genes [24].

The aim of this study was to investigate DNA methylation changes following n-3 FA supplementation in overweight and obese subjects and identify biological pathways potentially altered by the n-3 FA supplementation, by using whole-genome DNA methylation analyses. To our knowledge, this is the first study to examine the possible effect of n-3 FA supplementation on genome-wide DNA methylation levels in blood leukocytes of overweight and obese adults.

## Results

### Effects of n-3 FA supplementation

Biochemical parameters of study subjects (*n* = 36) before and after the n-3 FA supplementation are presented in Table 1. The supplementation was associated with a decrease in fasting plasma triglyceride (TG) concentrations, similar to results reported in full cohort [25]. In the same manner, total cholesterol (TC) and the ratio TC/high-density lipoprotein cholesterol (HDL-C) decreased whereas glucose concentrations slightly increased after the supplementation (Table 1). As expected, the supplementation was associated with a decrease in percentage and absolute values of linoleic, arachidonic, and total n-6 FAs (including all *cis* and *trans* n-6 FAs) in plasma phospholipids (*P* value <0.0001 for all, data not shown). It was also associated with an increase in percentage and absolute values of EPA, DHA, ratio n-3/n-6, and total n-3 FAs (including all *cis* and *trans* n-3 FAs) in plasma phospholipids (*P* value <0.0001 for all, data not shown).

**Table 1:** Biochemical parameters of subjects before and after n-3 FA supplementation (*n* = 36)

	Before n-3 suppl.	After n-3 suppl.	*P* value
Gender	18 men and 18 women		
Age (years)	34.7 ± 8.8		
BMI (kg/m^2^)	29.2 ± 3.65	29.2 ± 3.83	0.24
Triglycerides^a^ (mmol/L)	1.42 ± 0.80	1.24 ± 0.65	0.0034
Cholesterol (mmol/L)			
Total	5.24 ± 0.9	5.12 ± 0.92	0.048
LDL-C	3.18 ± 0.91	3.10 ± 0.92	0.19
HDL-C	1.40 ± 0.35	1.44 ± 0.40	0.061
Ratio TC/HDL-C	4.0 ± 1.31	3.85 ± 1.35	0.018
Glucose (mmol/L)	4.86 ± 0.51	5.06 ± 0.47	0.0085
Insulin^a^ (pmol/L)	108.0 ± 143.5	91.7 ± 48.4	0.70
CRP^a^ (mg/L)	3.89 ± 6.57	3.24 ± 5.32	0.18

Data are shown as mean ± SD

*Abbreviations*: *BMI* body mass index, *CRP* C-reactive protein, *HDL-C* high-density lipoprotein cholesterol, *LDL-C* low-density lipoprotein cholesterol, *suppl* supplementation, *TC* total cholesterol

^a^ *P* value derived from log_10_ transformed data

### Genome-wide DNA methylation analyses

Globally, 484,027 of the 485,577 probes (99.7%) on the array were detected with a detection *P* value ≤0.05. After n-3 FA supplementation, 308 CpG sites, assigned to 231 genes, were differentially methylated (false discovery rate (FDR)-corrected DiffScore ≥│13│~ *P* ≤ 0.05). A total of 286 CpG sites were hypermethylated (93%) and 22 were hypomethylated (7%) after supplementation as compared to levels before supplementation (Table 2). A total of 36.4% of significant differentially methylated CpG sites were located in gene bodies (Table 2). The genomic localization of CpG sites is summarized in Table 2. Detailed information about the 308 differentially methylated CpG sites is presented in Additional file 1.

**Table 2:** Summary of methylation results

	*n*
Probes	485 577
Number of probes detected (*P* ≤ 0.05)	484 027
Differentially methylated (FDR-corrected DiffScore ≥ǀ13ǀ)	308
Hypermethylated after n-3 FA supplementation	286
Gene body	107
3′-UTR	34
1st exon	4
5′-UTR	19
Promoter region^a^	50
Intergenic region	72
Hypomethylated after n-3 FA supplementation	22
Gene body	5
3′-UTR	0
1st exon	1
5′-UTR	5
Promoter region^a^	6
Intergenic region	5

Localization according to the first annotated transcript for each CpG site and provided for the Infinium HumanMethylation450 BeadChip

*Abbreviations*: *FDR* false discovery rate, *UTR* untranslated region, *TSS* transcription start site

^a^Promoter region includes TSS1500 and TSS200

### Relationship between CpG sites and surrounding SNPs

Using results from a recent GWAS done by our group in the same cohort [26], we tested potential relationship between pre-supplementation methylation levels and changes in methylation levels (∆methylation) of the 308 differentially methylated CpG sites and surrounding single-nucleotide polymorphisms (SNPs) (±1 kb). Single-nucleotide polymorphisms (SNPs) had an effect on pre-supplementation methylation levels of 15 CpG sites. Moreover, SNPs only affected ∆methylation of two CpG sites. Indeed, rs41286653 and rs899388 affected ∆methylation of cg02296904 located in the body of the gene *GAK* (chromosome 4). The SNP rs114329043 also affected ∆methylation of cg27270362 located in an intergenic region on chromosome 10. We can conclude that the potential effect of the n-3 FA supplementation on ∆methylation of the 308 CpG sites is due in very small proportion to surrounding SNPs.

### Pathway analyses

From the 231 differentially methylated genes following supplementation, Ingenuity Pathway Analysis (IPA) mapped 227 genes. IPA revealed 55 pathways that were significantly overrepresented (*P* ≤ 0.05) (detailed pathways are presented in the Additional file 2), but we focused on the 16 pathways related to CV health. Selected CV health-related pathways, associated *P* values, and differentially methylated genes identified in pathways are presented in Table 3. These selected pathways revealed relevant genes known to be related to inflammatory and immune response (*AKT3*, *ATF1*, *BAX*, *CASP6*, *DHRS9*, *FAS*, *PRKAG2*, *PRKCZ*, *PRKD3*, *PTEN*, *TRIM24*), lipid metabolism (*AKT3*, *IGFBP5*, *KLK6*, *NUDT3*, *PLCH1*, *PPP2RE5*, *PTEN*, *PTPN12*, *PRKAG2*, *PRKCZ*, *PRKD3*, *SLCO1B3*), type 2 diabetes (T2D) (*AKT3*, *PRKAG2*, *PRKCZ*, *PRKD3*), and CV signaling (*AKT3*, *HDAC4*, *PRKAG2*, *PRKCZ*, *PRKD3*).

**Table 3:** CV health-related overrepresented pathways identified from differential methylation analysis following an n-3 FA supplementation

IPA canonical pathways	*P* value	Differentially methylated genes
Tumoricidal function of hepatic natural killer cells^a^	0.0017	*BAX*, *CASP6*, *FAS*
RAR activation^a^	0.0035	*AKT3*, *DHRS9*, *PRKAG2*, *PRKCZ*, *PRKD3*, *PTEN*, *TRIM24*
VDR/RXR activation^b^	0.0078	*IGFBP5*, *KLK6*, *PRKCZ*, *PRKD3*
Fcγ receptor-mediated phagocytosis in macrophages and monocytes^a^	0.017	*AKT3*, *PRKCZ*, *PRKD3*, *PTEN*
D-myo-inositol-5-phosphate metabolism^b^	0.018	*NUDT3*, *PLCH1*, *PPP2R5E*, *PTEN*, *PTPN12*
Nitric oxide signaling in the cardiovascular system^c^	0.026	*AKT3*, *PRKAG2*, *PRKCZ*, *PRKD3*
IL-3 signaling^a^	0.033	*AKT3*, *PRKCZ*, *PRKD3*
PXR/RXR activation^b^	0.035	*AKT3*, *SLCO1B3*, *PRKAG2*
LPS-stimulated MAPK signaling^a^	0.035	*ATF1*, *PRKCZ*, *PRKD3*
NF-кB activation by viruses^a^	0.037	*AKT3*, *PRKCZ*, *PRKD3*
CCR5 signaling in nacrophages^a^	0.037	*FAS*, *PRKCZ*, *PRKD3*
Role of NFAT in cardiac hypertrophy^c^	0.038	*AKT3*, *HDAC4*, *PRKAG2*, *PRKCZ*, *PRKD3*
P2Y purigenic receptor signaling pathway^c^	0.040	*AKT3*, *PRKAG2*, *PRKCZ*, *PRKD3*
Cytotoxic T lymphocyte-mediated apoptosis of target cells^a^	0.040	*FAS*, *CASP6*
PI3K signaling in B lymphocytes^a^	0.044	*AKT3*, *ATF1*, *PRKCZ*, *PTEN*
Type II diabetes mellitus signaling^d^	0.049	*AKT3*, *PRKAG2*, *PRKCZ*, *PRKD3*

Pathways related to the following: ^a^Inflammatory and immune response (*n* = 9); ^b^Lipid metabolism (*n* = 3); ^c^Cardiovascular signaling (*n* = 3); ^d^Diabetes (*n* = 1)

### DNA methylation and biochemical parameters

We further investigated the possible relationship between ∆methylation of these 19 genes related to CV health and changes in the four biochemical parameters modified by the n-3 FA supplementation (∆TG, ∆TC, ∆TC/HDL-C, and ∆Glucose). As shown in Table 4, ∆methylation of cg00011856 (*IGFBP5*) was positively correlated with ∆TG (*r* = 0.39, *p* = 0.023) while ∆methylation of cg05655647 (*ATF1*) was negatively correlated with ∆TG (*r* = −0.35, *p* = 0.047). The ∆methylation of cg00011856 (*IGFBP5*) and cg24455383 (*AKT3*) were positively correlated with ∆TC/HDL-C (*r* = 0.36, *p* = 0.042 and *r* = 0.42, *p* = 0.016, respectively). The ∆methylation of cg15656521 (*HDAC4*) was positively correlated with ∆Glucose (*r* = 0.35, *p* = 0.043). There was no significant correlation with ∆TC. After adjustments for age, sex, and body mass index (BMI), the correlation between ∆methylation of cg00011856 and ∆TG remained significant (*r* = 0.42, *p* = 0.020) as well as the one between ∆methylation of cg24455383 and ∆TC/HDL-C (*r* = 0.40, *p* = 0.031).

**Table 4:** Significant correlations between changes in biochemical parameters and changes in methylation levels following an n-3 FA supplementation

CpG site ID (gene, position^a^)	∆Triglyceride	∆TC/HDL-C	∆Glucose
∆cg00011856 (*IGFBP5*, Chr2:217560946)	0.39 (0.023)^b^	0.36 (0.042)	–
∆cg05655647 (*ATF1*, Chr12:51157023)	-0.35 (0.047)	–	–
∆cg24455383 (*AKT3*, Chr1:243736307)	–	0.42 (0.016)^b^	–
∆cg15656521 (*HDAC4*, Chr2:239970617)	–	–	0.35 (0.043)

Results are *R* (*P* value)

*Abbreviations*: *HDL-C* high-density lipoprotein cholesterol, *TC* total cholesterol

^a^All positions are from the Genome Build 37

^b^Correlation remains significant after adjustments for age, sex, and body mass index

Using previous data on gene expression in the same cohort [27], we performed further analyses to test whether correlations between ∆methylation and changes in biochemical parameters may potentially be attributable to changes in expression levels (∆expression). We first correlated ∆methylation and ∆expression for these four genes (*ATF1*, *AKT3*, *HDAC4*, and *IGFBP5*). *ATF1* and *HDAC4* showed significant correlation between ∆methylation and ∆expression after adjustments for the effects of age, sex, and BMI (*r* = −0.44, *p* = 0.02 and *r* = 0.47, *p* = 0.01). We then correlated ∆expression of *ATF1* and *HDAC4* with ∆TG and ∆Glucose, respectively. However, we found no significant association.

## Discussion

The aim of this study was to investigate DNA methylation changes following n-3 FA supplementation and identify potentially altered biological pathways. We first observed that a 6-week supplementation with 3 g of n-3 FAs per day was associated with a decrease in n-6 FAs and an increase in n-3 FAs in plasma phospholipids of 36 overweight and obese adults. The n-3 FA supplementation also decreased plasma TG, TC, and the ratio TC/HDL-C, while increasing plasma glucose concentrations. The slight increase in glucose concentrations was similar to the one reported in the entire cohort [25]. Studies have reported conflicting results on the effect of n-3 FAs on blood glucose concentrations [28]. There is a large variability in plasma glucose response to n-3 FAs with effects varying from 1.61 mmol/L net reduction to 1.4 mmol/L net increase [29].

Using a genome-wide methylation analysis, we identified differences in CpG sites methylation levels following an n-3 FA supplementation. As previously mentioned, only few studies were conducted on the impact of n-3 FAs on DNA methylation in humans [19–24, 30]. Our results are in agreement with these studies suggesting that EPA and DHA can modulate DNA methylation levels. Moreover, we identified overrepresented pathways from differentially methylated genes. More precisely, pathway analysis revealed 16 overrepresented pathways related to CV health. These results are in line with previously reported effects of n-3 FAs and DNA methylation on CV health. Indeed, n-3 FAs have beneficial effects on CV risk factors [1–9], even if they are not associated with CV disease events [31]. Moreover, epidemiological studies have reported the association between global DNA methylation levels and prevalence of CV diseases [17, 18].

Among the 16 pathways related to CV health, nine were related to inflammatory and immune response which may be in line with potential anti-inflammatory effects of n-3 FAs, more particularly DHA [5, 32]. Studies have also reported the link between DNA methylation and inflammation. A recent study in the GOLDN study and the ENCODE consortium reported that higher erythrocyte total n-3 FAs was associated with lower cg01770232 methylation (*IL-6*) and lower plasma IL-6 concentration [30]. The hypomethylation of long-interspersed element-1 was also associated with higher serum vascular cell adhesion molecule-1 in elderly men [33]. Moreover, three pathways were related to lipid metabolism, which is in accordance with known hypotriglyceridemic effects of n-3 FAs [1]. Methylation levels of one CpG site in *APOE* and one in *ABCA1* were negatively associated with plasma TG and HDL-C levels, respectively, in the GOLDN study [34, 35]. DNA methylation is also implicated in the regulation of atherosclerosis. Indeed, genome-wide DNA methylation changes (mainly hypermethylation) occur during the onset and progression of atherosclerotic lesions in humans [36, 37]. A total of three pathways were related to cardiovascular signaling. Indeed, reported antiplatelet effects of n-3 FAs [38] are consistent with the overrepresentation of the nitric oxide signaling in the cardiovascular system pathway identified herein. Finally, one pathway was related to T2D, which may be in line with potential, but yet controversial, effects of n-3 FAs on glucose homeostasis [39]. Data mining analysis suggests a role of epigenetic factors in the pathogenesis of T2D [40]. Differential methylation profiles in pancreatic islets from T2D and non-diabetic subjects were also identified thus suggesting a role of DNA methylation in pathogenesis of T2D [41]. Moreover, blood DNA methylation of some CpG sites have been associated with blood glucose concentrations in an epigenome-wide association study [42]. All together, these finding suggest a possible link between n-3 FAs, DNA methylation, and CV risk factors.

Among differentially methylated genes, some were also of particular interest in the field of CV health. For example, a genetic variation in the *FAS* gene was associated with an increased occurrence of myocardial infarction in Japanese subjects [43]. Interestingly, *FAS* methylation levels were significantly associated with n-3 FA intakes in Yup’ik Alaska Native individuals [23]. Moreover, a total of four CpG sites showed correlations between changes in their methylation levels and changes in biochemical parameters following the supplementation. First, ∆methylation of cg15656521 in *HDAC4* gene was positively correlated with ∆glucose. *HDAC4* gene encodes for a histone deacetylase 4 that is a signal-dependent modulator of transcription with role in muscle differentiation and neuronal survival [44]. *HDAC4* methylation was inversely associated with n-3 FAs in whole blood of men [45]. *HDAC4* also downregulates *GLUT4* transcription in cultured adipocytes and fasting mice [46]. Interestingly, Benton et al. reported a robust inverse correlation between changes in fasting glucose and changes in methylation of *HDAC4* (cg26078407) in subcutaneous fat [47]. However, they did not test for association with cg15656521 since it was not differentially methylated in their cohort after gastric bypass surgery [47]. Second, ∆methylation of cg00011856 in *IGFBP5* gene was positively correlated with ∆TG (even after adjustments for age, sex, and BMI) and with ∆TC/HDL-C. *IGFBP5* gene encodes for insulin-like growth factor binding protein 5 [48]. Young patients with coronary heart disease have significantly higher serum IGFBP5 than age- and BMI-matched controls [49]. A study in arthritic rats demonstrated that EPA increases *IGFBP5* mRNA in the gastrocnemius muscle [50]. Another study found higher *IGFBP5* expression in the liver of beef fed with n-3 FAs compared to control diet [51]. Unfortunately, we cannot verify if the n-3 FA supplementation increases *IGFBP5* expression in our sample since it is not expressed in blood [52], but it would be interesting to look at its expression in the liver after an n-3 FA supplementation. Third, ∆methylation of cg24455383 in *AKT3* was positively correlated with ∆TC-HDL-C, even after adjustments. *AKT3* gene encodes for an AKT serine/threonine kinase 3 stimulated by insulin and growth factors [53, 54]. It is likely involved in insulin-stimulated glucose transport in the human skeletal muscles [55]. *AKT3* also has anti-atherogenic properties due to its capacity to inhibit macrophages foam cells formation by reducing lipoprotein uptake and promoting ACAT-1 degradation [56, 57]. AKT signaling has also been shown to regulate lipid metabolism through phosphorylation and inhibition of GSK3 [58]. The association between changes in *AKT3* methylation and changes in TC/HDL-C ratio reported herein may be plausible since *AKT3* seems to be involved in lipid metabolism. Finally, ∆methylation of cg05655647 in *ATF1* gene was negatively correlated with ∆TG. The *ATF1* gene encodes for the activating transcription factor 1 that leads to the production of atheroprotective macrophages [59, 60]. SNPs within this gene were also associated with an increased risk of essential hypertension in a case-control study [61]. Data are insufficient to propose a mechanism of action between methylation of *ATF1* and plasma TG levels. Nevertheless, we could hypothesize that methylation of these genes may play a role in the effects of n-3 FAs on CV health since they are modulated by n-3 FAs, they have been associated with CV health in pathway analysis, and they are correlated with changes in biochemical parameters. However, we acknowledge that our study design does not allow us to prove or investigate causality between n-3 FA supplementation, DNA methylation, and CV disease risk factors. Moreover, we were not able to investigate the potential link between ∆methylation of *ATF1*, *AKT3*, *IGFBP5*, and *HDAC4* and changes in biochemical parameters using ∆expression. At this time, we cannot rule out the possibility that the small sample size or low ∆expression values of these genes limit our statistical power to detect significant associations. This possible link does not seem to be due either to surrounding SNPs since they affected ∆methylation of only two CpG sites among the 308 CpG sites.

The present study has some limitations. DNA methylation levels are specific to the type of cell and tissue [62]. These methylation profiles in blood leukocytes might not represent DNA methylation in other tissues even though these patterns are globally conserved across tissues [63–65]. Pathway analysis has certain methodological considerations. As part of Genetic Analysis Workshop 18, seven research groups raised the fact that annotation of genetic variants is inconsistent across databases, incomplete and biased toward known genes [66]. Moreover, insufficient statistical power is an issue in pathway analyses [66]. Thus, these results need to be validated in larger and independent studies considering that replication remains the gold standard to establish validity of the findings. Finally, the sample size is relatively small so these results need to be validated in larger, independent studies.

## Conclusions

In conclusion, the present data provide key differences in blood leukocytes DNA methylation levels of subjects following n-3 FA supplementation, which provides new, potential insights on novel genes and metabolic pathways underlying the effects of n-3 FAs on the CV risk profile. Further studies in larger, independent samples are required to unveil potential functional mechanisms underlying metabolic improvements with n-3 FA supplementation.

## Methods

### Study population and study design

The present study is based on a subsample of a larger intervention study that aimed at studying the inter-individual variability in TG response to an n-3 FA supplementation as previously described [2]. A total of 254 subjects from the greater Quebec City metropolitan area (Canada) were recruited between September 2009 and December 2011. In total, 210 subjects completed the intervention. Non-smoker subjects, aged between 18 and 50, with a BMI between 25 and 40 kg/m^2^, and not taking any lipid-lowering medication were included. Subjects were excluded if they had taken n-3 FA supplementation at least 6 months prior to the intervention or had been diagnosed with any metabolic disorder. A subset of 36 subjects who first completed the study (equal proportion of men and women) was selected for the purpose of this genome-wide DNA methylation analysis.

First, subjects received dietary instructions by a trained registered dietician to achieve recommendations from Canada’s Food Guide during a 2-week run-in period [67]. After this period, subjects receive a bottle containing fish oil capsules needed for the following 6 weeks. They were invited to take five capsules per day providing a total of 3 g of n-3 FAs (including 1.9–2.2 g of EPA and 1.1 g of DHA) per day. Compliance was assessed by the count of remaining capsules and by measuring EPA and DHA in plasma phospholipids. Details on the study design have been described elsewhere [2]. The experimental protocol was approved by the Ethics Committees of Laval University Hospital Research Center and Laval University. This trial was registered at clinicaltrials.gov as NCT01343342.

### Anthropometric and metabolic measurements

Body weight, waist girth, and height were measured according to the procedures recommended by the Airlie Conference [68] and were taken before the run-in period as well as before and after the supplementation period. Blood samples were collected from an antecubital vein into vacutainer tubes containing EDTA after 12-h overnight fast and 48-h alcohol abstinence before the run-in period to identify and exclude participants with metabolic disorders. Afterwards, the selected participants had blood samples taken before and after the supplementation period. Plasma was separated by centrifugation (2500 g for 10 min at 4 °C), and samples were aliquoted and frozen (−80 °C) for subsequent analyses. Enzymatic assays were used to measure plasma TC and TG concentrations [69, 70]. Precipitation of very-low density lipoprotein (VLDL) and low-density lipoprotein (LDL) particles in the infranatant with heparin manganese chloride generated the HDL-C fraction [71]. LDL cholesterol (LDL-C) was calculated with the Friedewald formula [72]. Using a sensitive assay, plasma C-reactive protein (CRP) was measured by nephelometry (Prospec equipment Behring) [73].

### DNA extraction and DNA methylation analysis

We extracted genomic DNA from blood leukocytes using the GenElute Blood Genomic DNA kit (Sigma-Aldrich, St. Louis, MO, USA) for the 72 samples: 36 samples before and 36 samples after the supplementation. DNA was quantified using both NanoDrop Spectrophotometer (Thermo Scientific, Wilmington, DE, USA) and PicoGreen DNA methods. McGill University and Genome Quebec Innovation Center (Montreal, QC, Canada) conducted the bisulfite conversion and quantitative DNA methylation analysis using Infinium HumanMethylation450 array (Illumina, San Diego, CA, USA). Three samples (one sample before and two samples after supplementation) were excluded from microarray analysis following quality control steps (bisulfite conversion, extension, staining, hybridization, target removal, negative and nonpolymorphic control probes).

We used Illumina GenomeStudio software version 2011.1 and the Methylation Module to analyze methylation data on 485,577 CpG sites. Methylation levels (varying from 0 to 1) were estimated as the proportion of total signal intensity from methylated-specific probe. To avoid false positives, probes with a detection *P* value >0.05 in more than 10% of samples were removed. Probes on the X and Y chromosomes were also removed to eliminate gender bias. Thus, 472,715 probes were considered in differential methylation analysis. Differences in methylation levels before and after n-3 FA supplementation were tested using the Illumina Custom model in GenomeStudio software. FDR-corrected DiffScores were computed to account for multiple testing and limit false positives. We established significant differences following n-3 FA supplementation with a FDR-corrected DiffScore ≥│13│~ *P* ≤ 0.05.

### Pathway analyses

We used the IPA system (Ingenuity® Systems, www.ingenuity.com) to analyze potentially modified pathways from differentially methylated genes following the supplementation. Using a right-tailed Fisher’s exact test, IPA measured the likelihood that pathways were overrepresented among the list of significant differentially methylated genes.

### Statistical analyses

We tested potential relationship between pre-supplementation methylation levels and ∆methylation of the 308 CpG sites and surrounding SNPs (±1 kb). We used results from a recent GWAS done by our group in the same cohort (*n* = 141) [26]. We considered only SNPs located at ±1 kb from CpG sites and with a minor allele frequency ≥1%. We tested 652 associations using analysis of variance (general linear model, type III sum of squares) and adjusted for the effects of age, sex, and BMI. We used a Bonferroni correction for multiple testing; thus, associations with a *P* value ≤ 7.67 × 10^−5^ (0.05/652) were considered significant.

Biochemical parameters are expressed as means ± SD. We used a paired Student’s *t* test to test differences in biochemical parameters before and after the supplementation. Variables not normally distributed were log_10_ transformed before analyses. ∆methylation of CpG sites (*n* = 20) within genes in the 16 pathways related to CV health were defined as post-supplementation methylation levels minus pre-supplementation methylation levels. Changes in the four significantly modulated biochemical parameters (∆TG, ∆TC, ∆TC/HDL-C, and ∆Glucose) were defined as (post-supplementation values minus pre-supplementation values)/ pre-supplementation values to account for baseline values. Potential relationships between ∆methylation, ∆expression, and changes in biochemical parameters (∆TG, ∆TC, ∆TC/HDL-C, and ∆Glucose) were investigated using Pearson’s correlation. We also accounted for potential confounding effects of age, sex, and BMI in correlations. Statistical analyses were conducted using SAS software version 9.3 (SAS Institute, Cary, NC, USA).

## Additional files

Additional file 1: Differentially methylated probes after the n-3 FA supplementation. Description of data: List of all 308 differentially methylated probes (FDR-corrected DiffScore ≥│13│~ *P* ≤ 0.05). (XLSX 42 kb)

Additional file 2: Overrepresented pathways identified from differential methylation analysis following an n-3 FA supplementation. Description of data: Table describing all 55 significant overrepresented pathways identified from methylation analysis (IPA canonical pathways, associated *P* value, and list of differentially methylated genes). (DOCX 21 kb)

## Abbreviations

BMI: Body mass index
CpG: Cytosine-phosphate-guanine
CV: Cardiovascular
DHA: Docosahexaenoic acid
EPA: Eicosapentaenoic acid
FDR: False discovery rate
HDL-C: High-density lipoprotein cholesterol
IPA: Ingenuity Pathway Analysis
n-3 FAs: Omega-3 fatty acids
SNPs: Single-nucleotide polymorphisms
T2D: Type 2 diabetes
TC: Total cholesterol
TG: Triglyceride
