# Supplementary material for: Epigenetic changes in blood leukocytes following an omega-3 fatty acid supplementation
Source: Clin Epigenetics. 2017 Apr 26;9:43. doi: 10.1186/s13148-017-0345-3 (PMC5405524; doi:10.1186/s13148-017-0345-3)
Supplement: Supplementary file 2 — Overrepresented pathways identified from differential methylation analysis following an n-3 FA supplementation. Description of data: Table describing all 55 significant overrepresented pathways identified from methylation analysis (IPA canonical pathways, associated P value, and list of differentially methylated genes). (DOCX 21 kb) [file 13148_2017_345_MOESM2_ESM.docx]

| **Additional file 2.** Overrepresented pathways identified from differential methylation analysis following an n-3 FA supplementation | | |
| --- | --- | --- |
| IPA Canonical Pathways | *P* value | Differentially methylated genes |
| Ovarian Cancer Signaling | 0.00036 | *AKT3, FGF9, GJA1, PRKAG2, PTEN, RB1, TFDP1* |
| p53 Signaling | 0.00044 | *AKT3, BAX, CASP6, FAS, PTEN, RB1* |
| Glioma Signaling | 0.00048 | *AKT3, PRKCZ, PRKD3, PTEN, RB1, TFDP1* |
| 14-3-3-mediated Signaling | 0.0012 | *AKT3, BAX, PRKCZ, PRKD3, SRPK2, VIM* |
| Tumoricidal Function of Hepatic Natural Killer Cells | 0.0017 | *BAX, CASP6, FAS* |
| Myc Mediated Apoptosis Signaling | 0.0026 | *AKT3, BAX, FAS, PRKCZ* |
| RAR Activation | 0.0035 | *AKT3, DHRS9, PRKAG2, PRKCZ, PRKD3, PTEN, TRIM24* |
| Small Cell Lung Cancer Signaling | 0.0063 | *AKT3, PTEN, RB1, TFDP1* |
| Tight Junction Signaling | 0.0065 | *AKT3, CPSF6, PPP2R5E, PRKAG2, PRKCZ, PTEN* |
| Cyclins and Cell Cycle Regulation | 0.0076 | *HDAC4, PPP2R5E, RB1, TFDP1* |
| VDR/RXR Activation | 0.0078 | *IGFBP5, KLK6, PRKCZ, PRKD3* |
| Melanoma Signaling | 0.0093 | *AKT3, PTEN, RB1* |
| Ceramide Signaling | 0.0098 | *AKT3, CERK, PPP2R5E, PRKCZ* |
| Prostate Cancer Signaling | 0.010 | *AKT3, PTEN, RB1, TFDP1* |
| Molecular Mechanisms of Cancer | 0.012 | *AKT3, BAX, CASP6, FAS, PRKAG2, PRKCZ, PRKD3, RB1, TFDP1* |
| Axonal Guidance Signaling | 0.013 | *ADAM2, ADAM28, AKT3, EFNA5, EPHA10, PRKAG2, PRKCZ, PRKD3, SRGAP1, WIPF1* |
| Neuregulin Signaling | 0.014 | *AKT3, PRKCZ, PRKD3, PTEN* |
| Chronic Myeloid Leukemia Signaling | 0.014 | *AKT3, HDAC4, RB1, TFDP1* |
| G Beta Gamma Signaling | 0.016 | *AKT3, PRKAG2, PRKCZ, PRKD3* |
| Fcγ Receptor-mediated Phagocytosis in Macrophages and Monocytes | 0.017 | *AKT3, PRKCZ, PRKD3, PTEN* |
| Telomerase Signaling | 0.017 | *AKT3, HDAC4, PPP2R5E, RB1* |
| IGF-1 Signaling | 0.017 | *AKT3, IGFBP5, PRKAG2, PRKCZ* |
| D-myo-inositol-5-phosphate Metabolism | 0.018 | *NUDT3, PLCH1, PPP2R5E, PTEN, PTPN12* |
| Neuropathic Pain Signaling In Dorsal Horn Neurons | 0.020 | *GPR37, PRKAG2, PRKCZ, PRKD3* |
| Estrogen-mediated S-phase Entry | 0.023 | *RB1, TFDP1* |
| Cell Cycle: G1/S Checkpoint Regulation | 0.026 | *HDAC4, RB1, TFDP1* |
| Nitric Oxide Signaling in the Cardiovascular System | 0.026 | *AKT3, PRKAG2, PRKCZ, PRKD3* |
| Huntington's Disease Signaling | 0.030 | *AKT3, BAX, CASP6, HDAC4, PRKCZ, PRKD3* |
| Dopamine-DARPP32 Feedback in cAMP Signaling | 0.030 | *ATF1, PPP2R5E, PRKAG2, PRKCZ, PRKD3* |
| Ephrin Receptor Signaling | 0.032 | *AKT3, EFNA5, EPHA10, SORBS1, WIPF1* |
| Erythropoietin Signaling | 0.032 | *AKT3, PRKCZ, PRKD3* |
| IL-3 Signaling | 0.033 | *AKT3, PRKCZ, PRKD3* |
| Non-Small Cell Lung Cancer Signaling | 0.033 | *AKT3, RB1, TFDP1* |
| PEDF Signaling | 0.033 | *AKT3, FAS, TCF12* |
| PXR/RXR Activation | 0.035 | *AKT3, SLCO1B3, PRKAG2* |
| p70S6K Signaling | 0.035 | *AKT3, PPP2R5E, PRKCZ, PRKD3* |
| LPS-stimulated MAPK Signaling | 0.035 | *ATF1, PRKCZ, PRKD3* |
| NF-кB Activation by Viruses | 0.037 | *AKT3, PRKCZ, PRKD3* |
| CCR5 Signaling in Macrophages | 0.037 | *FAS, PRKCZ, PRKD3* |
| Role of NFAT in Cardiac Hypertrophy | 0.038 | *AKT3, HDAC4, PRKAG2, PRKCZ, PRKD3* |
| Sertoli Cell-Sertoli Cell Junction Signaling | 0.038 | *AKT3, PLS1, PRKAG2, PTEN, SORBS1* |
| PI3K/AKT Signaling | 0.039 | *AKT3, PPP2R5E, PRKCZ, PTEN* |
| Glutamate Dependent Acid Resistance | 0.039 | *GAD1* |
| P2Y Purigenic Receptor Signaling Pathway | 0.040 | *AKT3, PRKAG2, PRKCZ, PRKD3* |
| Melatonin Signaling | 0.040 | *PRKAG2, PRKCZ, PRKD3* |
| HER-2 Signaling in Breast Cancer | 0.040 | *AKT3, PRKCZ, PRKD3* |
| Cytotoxic T Lymphocyte-mediated Apoptosis of Target Cells | 0.040 | *FAS, CASP6* |
| Hereditary Breast Cancer Signaling | 0.042 | *AKT3, HDAC4, PTEN, RB1* |
| PI3K Signaling in B Lymphocytes | 0.044 | *AKT3, ATF1, PRKCZ, PTEN* |
| mTOR Signaling | 0.044 | *AKT3, PPP2R5E, PRKAG2, PRKCZ, PTEN* |
| D-myo-inositol (1,4,5,6)-Tetrakisphosphate Biosynthesis | 0.047 | *NUDT3, PPP2R5E, PTEN, PTPN12* |
| D-myo-inositol (3,4,5,6)-tetrakisphosphate Biosynthesis | 0.047 | *NUDT3, PPP2R5E, PTEN, PTPN12* |
| Cell Cycle Regulation by BTG Family Proteins | 0.047 | *PPP2R5E, RB1* |
| VEGF Family Ligand-Receptor Interactions | 0.048 | *AKT3, PRKCZ, PRKD3* |
| Type II Diabetes Mellitus Signaling | 0.049 | *AKT3, PRKAG2, PRKCZ, PRKD3* |
